# XEN45 Gel Stent Combined with Healaflow Injectable Viscoelastic Implant

**DOI:** 10.1155/2023/7096406

**Published:** 2023-11-22

**Authors:** Eloy Villarreal, Eran Berkowitz, Beatrice Tiosano

**Affiliations:** Department of Ophthalmology, Hillel Yaffe Medical Center Affiliated with the Bruce Rappaport School of Medicine, The Technion-Israel Institute of Technology, Haifa, Israel

## Abstract

**Purpose:**

To introduce a potential solution for failed glaucoma surgeries by proposing an optional surgical procedure in conjunction with the use of Healaflow (Anteis S.A., Geneva, Switzerland) as a spacer, which may potentially reduce the failure rate. *Case Presentation*. We present the outcomes of a surgical procedure involving the inferonasal implantation of an ab interno XEN gel stent (Allergan, Dublin, Ireland) in a 74-year-old male patient who was experiencing uncontrolled advanced glaucoma in his left eye. It is important to note that the patient had previously undergone several glaucoma surgeries and procedures in the same eye. During this particular procedure, we utilized Healaflow as a spacer by implanting the stent within a subconjunctival Healaflow “bubble.” At 6 months postoperatively, intraocular pressure remained on target. There was no need for additional topical medications, and no change in visual acuity was observed.

**Conclusion:**

For patients with a history of unsuccessful glaucoma surgeries and who are unsuitable candidates for tube shunt procedures or transscleral diode cyclophotocoagulation, an alternative option involves implanting the XEN45 stent in the inferior nasal region in conjunction with the use of subconjunctival Healaflow. This combined approach may provide a potential solution for managing glaucoma in these patients.

## 1. Introduction

Glaucoma, an ocular disease, is characterized by damage to the visual field and the deterioration of the optic nerve. The primary manifestation of this condition is the abnormal elevation of intraocular pressure (IOP). In recent developments, various minimally invasive glaucoma surgery (MIGS) devices have been created, allowing for implantation from within the eye (ab interno). This approach is aimed at providing a safer and less invasive method of lowering IOP compared to conventional surgery [1]. One of these devices, the XEN45 gel implant (XEN45) (Allergan, Dublin, Ireland), establishes a lasting drainage route from the anterior chamber to the subconjunctival space. This innovative approach circumvents the often obstructed natural drainage pathways observed in glaucoma patients [2–4].

The XEN45 gel stent is typically placed in the superonasal quadrant to preserve the superior conjunctival area, often chosen for a trabeculectomy. However, in certain instances, even with successful placement, the filtering bleb can extend into the superior region, potentially causing changes in the conjunctiva that could hinder future trabeculectomies. Therefore, there can be a consideration for implanting the XEN45 gel stent in the inferonasal quadrant instead. This approach would leave the entire superior area untouched, making future trabeculectomies more straightforward. It would also enhance accessibility during surgery, especially when dealing with patients who have prominent cheekbones, which can sometimes impede access to the superior quadrant during stent insertion. In situations where previous glaucoma surgeries have failed, resulting in scarring or ischemia in the superior conjunctiva, the logical progression is to consider a glaucoma drainage device (GDD) or transscleral diode cyclophotocoagulation (CPC). However, in selected cases, placing an XEN45 gel stent in an inferior location could be a viable alternative. This approach is less invasive, easier to perform, quicker to apply, and associated with a lower risk of corneal endothelial cell loss when compared to GDD or cyclodestructive procedures [5].

Several complications have been associated with the XEN45 gel stent implantation. The most feared postoperative complication is endophthalmitis. Endophthalmitis associated with XEN procedures is linked with several factors, including subsequent postoperative interventions, exposure of the bleb, defects in the conjunctiva, and the extended use of postoperative antibiotics [6]. However, there is still more to learn as we gain more experience with this device. Currently, all studies reporting on the increased risk of endophthalmitis due to an inferior filtering bleb have been associated with a trabeculectomy. An inferior trabeculectomy placement has been found to incur a 7-fold risk for endophthalmitis compared to a superior location [7].

Healaflow is an innovative form of a cross-linked sodium hyaluronate product [8] designed to be gradually absorbed by the human body. Its anti-inflammatory properties generate an antiscarring effect postglaucoma surgery. Healaflow has been shown to be effective in deep sclerectomy [8] and trabeculectomy [9]. Nevertheless, there remains a lack of pertinent clinical evidence supporting its utilization, especially in the new generation of minimally invasive bleb surgeries (MIBS) such as the XEN45 implant.

The authors have completed the CARE Checklist for this case report.

## 2. Case Presentation

**Figure 1:**
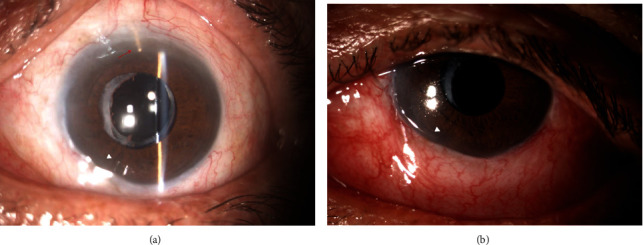
(a) Superior express shunt (red arrow) with adjacent superior scarred conjunctiva. Inferonasal XEN stent (white triangle). (b) Inferonasal XEN stent (white triangle) with inferior elevated functional bleb.

A 74-year-old male suffering from advanced pseudoexfoliation (PXF) glaucoma presented to our glaucoma unit with uncontrolled IOP (28 mmHg) in his left eye. The patient had previously undergone glaucoma surgeries and procedures in the left eye (Express Shunt, SLT, Micropulse CPC) (Figure 1(a)); however, his IOP when admitted to the ER was above target, despite maximal tolerated medical therapy (MTMT). Moreover, his visual fields were deteriorating; therefore, in consultation with the patient, it was decided to undergo XEN implantation in the left eye. Since the superior part the patient's conjunctiva was immobile and partially scarred, during the surgery, the XEN gel stent was placed inferiorly (Figure 1(b)), where the conjunctiva was healthy-looking and mobile.

### 2.1. Surgical Procedure

XEN implantation was performed as an independent procedure using a standardized implantation technique. To provide a concise overview, the XEN45 (Allergan, Dublin, Ireland) was inserted under intracameral anesthesia via an ab interno approach. The procedure commenced with a subconjunctival injection of 0.1 ml of Mitomycin C (MMC) (Lapidot Group Pharma, Caesarea, Israel) solution, consisting of 0.02% MMC with a total dose of 10 *μ*g, administered in the nasal inferior quadrant to prevent conjunctival scarring. Subsequently, 0.1 ml of Healaflow (Swiss Aptissen) was also injected subconjunctivally, positioned between the limbus and the previously injected MMC. Following the filling of the anterior chamber with a medium-grade viscoelastic substance (Hanita Visco 1.8%, Lapidot Group Pharma, Caesarea, Israel), the preloaded XEN injector needle was introduced through a 2.4 mm corneal paracentesis incision located superotemporally. The needle was then carefully guided across the anterior chamber, and the XEN device was implanted, ensuring that its subconjunctival portion was positioned within the Healaflow bleb. Finally, the eye was left partially filled with a viscoelastic material.

### 2.2. Outcomes

On the first day postoperatively, the intraocular pressure (IOP) significantly dropped to 7 mmHg. However, during the subsequent follow-up visits, the patient experienced hypotony in conjunction with the development of choroidals, which was effectively addressed through conservative management involving the use of topical medications.

By the six-month postoperative mark, the IOP had stabilized at 14 mmHg. There was an elevated inferior bleb, the choroidals had completely resolved, and the patient no longer required additional topical medications.

This study protocol was approved by the local Institutional Review Board and Ethics Committee of the Hillel Yaffe Medical Center, Israel, approval number 220822.

## 3. Discussion

Healaflow is an isotonic colloidal liquid composed of sodium hyaluronate with a cross-network structure. When injected subconjunctivally, it offers several potential advantages [10]. The structural composition and isotonic properties of Healaflow contribute to maintaining a steady and uniform flow of the aqueous humor. Furthermore, the viscoelastic properties can also assist in the XEN insertion, by dissecting the subconjunctival space, thus facilitating XEN implantation and reducing needling rates [11]. Most importantly, Healaflow can reduce the risk of XEN implant exposure and extrusion, acting as a barrier, thus subsequently reducing the risk of serious complications, i.e., endophthalmitis.

Healaflow is slowly resorbable, conveying a protective effect for a relatively long period of time (as indicated in earlier studies, it typically undergoes complete degradation in the body within a period of ~3 months). Several studies have further demonstrated that sodium hyaluronate can suppress the expression of cytokines and inflammatory factors, which, in turn, reduces the release of free radicals, thus decelerating the scarring and fibrosis processes associated with filtration surgery [12]. A recent study [9] comparing a trabeculectomy with and without the use of Healaflow showed no short-term (3 months) differences; however, at the 6-month follow-up, the Healaflow group showed a higher rate of functional filtration blebs and less scarring formation. Typically, the XEN45 gel stent is implanted in the superonasal region. Nevertheless, placing it in the nasally inferior quadrant could fully preserve the superior region for potential future trabeculectomies or alternatively, as in our case, add an optional location for implantation if the superior part of the conjunctiva is scarred or unfit for bleb-dependent procedures. Previous studies have demonstrated that inferior nasal implantation of XEN is as safe and effective as superior nasal implantation [13, 14].

Traditionally, GDD implantation or CPC is the preferred surgical approach in patients who had experienced a previous failed trabeculectomy. In the case of our patient, we believed that opting for XEN implantation could serve as a favorable substitute for conventional surgical interventions. This consideration stemmed from the potential endothelial cell loss resulting from the patient's prior recurrent surgeries. Furthermore, the decision for a GDD or CPC procedure would pose a higher risk of corneal decompensation [5, 15]. In addition, the patient's clinical state prevented him from undergoing lengthy surgeries. This is the first published description of Healaflow used with XEN implantation. Our experience has demonstrated that it can be a beneficial and an effective addition to the procedure, preventing short-term scarring and fibrosis.

In summary, the placement of the XEN45 stent in the inferior nasal region should be regarded as a viable choice for patients with a history of prior glaucoma surgeries, a heightened susceptibility to endothelial cell loss, and significant scarring in the superior conjunctival area and for cases where tube shunt procedures or CPC is not feasible. Furthermore, the incorporation of subconjunctival Healaflow into the XEN implantation procedure may potentially decrease failure rates and complications [16].

## Data Availability

All data generated or analyzed during this study are included in this article. Further enquiries can be directed to the corresponding author.
